# Uncovering Allosteric Signaling Pathways of GLP-1R via Molecular Dynamics and Network Theory

**DOI:** 10.21203/rs.3.rs-10245055/v1

**Published:** 2026-07-23

**Authors:** Long Tran, Zhijun Li, Preston Moore

**Affiliations:** Saint Joseph’s University, 5600 City Ave, Philadelphia, PA 19131

**Keywords:** GLP-1R, GPCR, molecular dynamics, network theory, allostery, meta paths

## Abstract

The glucagon-like peptide-1 receptor (GLP-1R) is a class B1 G protein-coupled receptor (GPCR) and an important therapeutic target for type 2 diabetes and obesity. While structural studies have identified binding sites on GLP-1R for agonists and allosteric modulators, the residue-level communication pathways underlying receptor activation and inhibition, as well as allosteric regulation, are not completely understood. Here, we performed 3-μs molecular dynamics simulations of GLP-1R in its apo state and when independently bound to a positive allosteric modulator (PAM), a negative allosteric modulator (NAM), and to agonists such as glucagon-like peptide-1 (GLP-1) and the stimulatory G protein (Gs). The resulting trajectories were analyzed using root-mean square deviation (RMSD) analysis and protein structure network (PSN) theory.

Comparison of the dynamic structural changes in GLP-1R, such as in its conserved HETx motif and polar networks in the transmembrane domain (TMD) in particular, revealed distinct communication pathways associated with receptor activation and inhibition. Negative effectors, such as NAM NNC0640, reinforced interactions within the central polar network, whereas positive effectors, like PAM C-1 or GLP-1 with Gs, favored signaling through TM3/TM5 or TM6/TM7 pathways, respectively. Across all systems, R190^2.60b^ emerged as a highly recurrent communication hub. These results identify residue-level pathways that may contribute to receptor activation and inhibition and suggest that positive and negative modulators reshape the allosteric communication network of GLP-1R in distinct ways.

## Introduction

GLP-1R belongs to the Class B1 secretin family of GPCRs and is critical in reducing blood sugar levels and regulating metabolism [1]. Like other GPCRs, it also has 7 helices that transverse a membrane bilayer, which gives rise to its TMD. On either side of its bilayer, GLP-1R also has an extracellular domain (ECD), where a ligand can bind to and function as an agonist or an antagonist to activate or deactivate GLP-1R, respectively, and an intracellular domain (ICD) where a G protein or other molecule can bind for downstream signaling.

Conventionally, the activation of GLP-1R at its orthosteric site begins with the binding of an agonist, such as GLP-1. With GLP-1, its C-terminal binds to the ECD of GLP-1R and its N-terminal then binds to the TMD, and this is often referred to as the two-domain model [2]. This prompts the subsequent binding of Gs at the ICD and this leads to the production of cyclic AMP (cAMP), which can trigger a reaction that results in the secretion of insulin to lower blood glucose levels. Conversely, the binding of an antagonist at the orthosteric site instead stabilizes the inactive conformation of GLP-1R.

In recent years, it has been reported that small-molecules that function as PAMs or NAMs can regulate GLP-1R activity through binding to an allosteric site. The PAM in our study, C-1, has been shown to successfully activate GLP-1R via cAMP assays, even without GLP-1 being bound to the ECD, so it is also considered an ago-PAM since it can act as an agonist [3]. Like other agonists, C-1 is assumed to cause TM6 to rotate outward, which is a hallmark of GLP-1R activation, and this creates space for the Gs to bind [4].

The NAM used in our simulations, NNC0640, binds to S352^6.41b^ and N406^8.47b^ of GLP-1R, which is also the same residues that C-1 binds to, and the main difference is that NNC0640 also binds to T355^6.44b^ to inactivate GLP-1R. [5]. The residue naming scheme used throughout this study corresponds to the Wooten numbering system and denotes its location on GLP-1R and relative position to the most conserved residue [6].

**Fig. 1:**
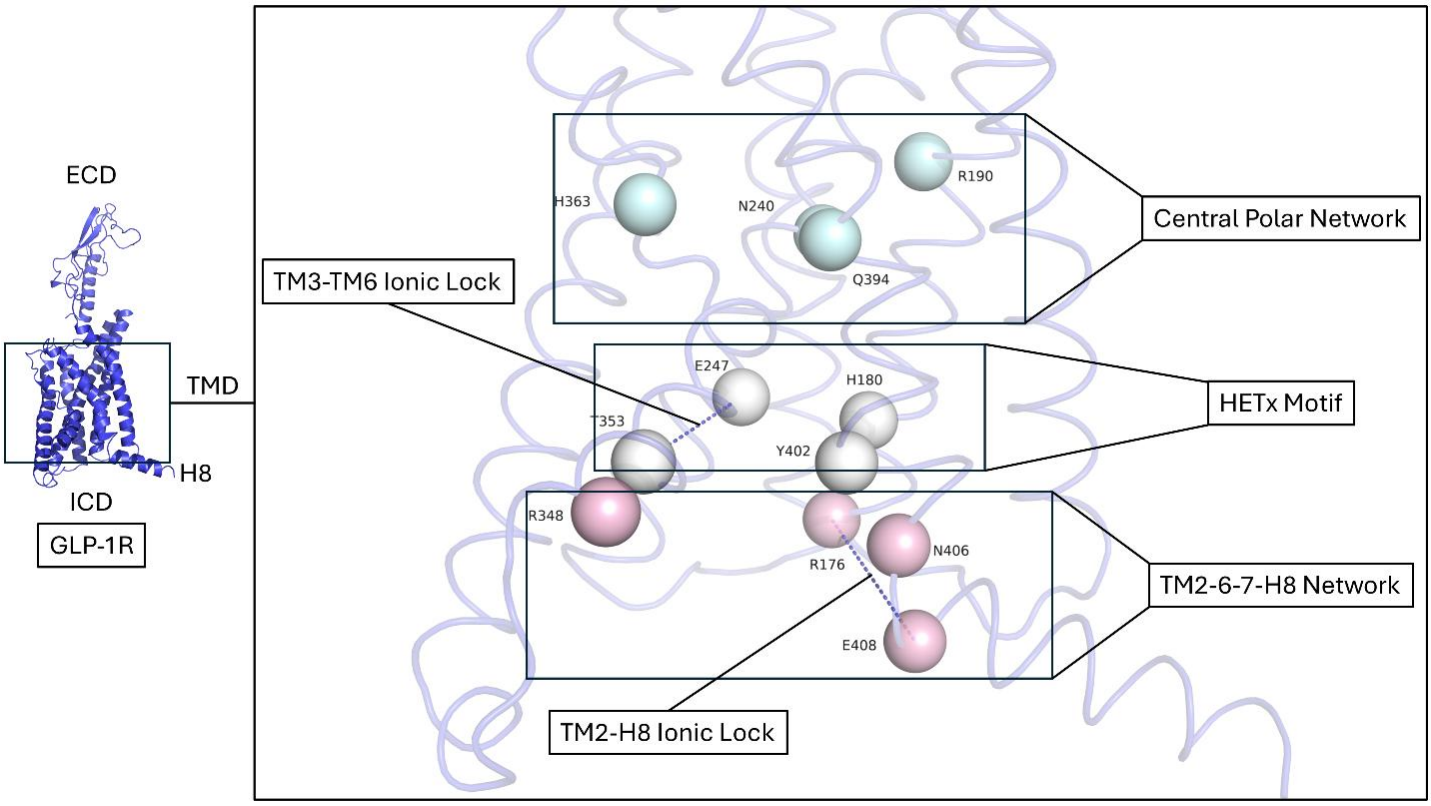
Conserved motifs and interaction networks in the TMD of GLP-1R. Residues of the central polar network (light cyan) accepts GLP-1 upon binding. The HETx motif (white) contains the TM3-TM6 ionic lock and is below the central polar network. The TM2–6-7-H8 network (light pink) is located at the cytoplasmic end and contains the TM2-H8 ionic lock

Within its TM domain, GLP-1R contains several conserved motifs and polar networks that are essential to its activation (Fig. 1). First, the central polar network (R190^2.60b^, N240^3.43b^, H363^6.52b^, Q394^7.49b^) below the ECD plays an important role in the activation of GLP-1R (Fig. 1) when an agonist is bound [7]. Below this central polar network is the conserved HETx motif (H180^2.50b^, E247^3.50b^, T353^6.42b^, Y402^7.57^). The disruption of the TM3-TM6 ionic lock within it can create more distance between those helices for Gs to bind and activate GLP-1R. Another polar network known as the TM2–6-7-H8 polar network (R176^2.46b^, R348^6.37b^, N406^8.47b^, E408^8.49b^) is located below the HETx motif and can aid in GLP-1R activation also if the TM2-H8 ionic lock is disrupted [8].

Network theory has been a crucial tool in recent years for the analysis of biological systems, including those within molecular dynamic trajectories [9]. It is derived from graph theory and can illustrate how components in systems are connected based on their link recurrence and frequencies. By assigning nodes and edges as residues and pairs, respectively, protein structure networks can be created from a structure, or a trajectory of structures in this case, and the difference between each system can be evaluated.

While binding sites of positive and negative allosteric modulators have been structurally characterized, the residue-level communication pathways through which these ligands modulate receptor activity remain poorly understood. The first full- length structure of GLP-1R was determined by cryo-EM [10] and was adopted as a starting point. We then created MD systems and combined the results from multi-microsecond molecular dynamics simulations with protein structure network analysis to determine how positive and negative modulators reshape allosteric signaling in GLP-1R.

## Methods

### Creating the molecular dynamic systems

The structure and positions for GLP-1R, GLP-1, and Gs used in our simulations were obtained from PDB:5VAI [10]. To refine the loops present in the GLP-1R structure, we used Prime from Schrodinger’s homology modeling software (version 2018–3) and performed extended loop refinement [11]. Positions of all components were retained from the PDB structure. We obtained the complex structure of GLP-1R with C-1 from our collaborators, which was obtained through molecular docking and confirmed by site specific mutagenesis studies [3].

The complex structure of GLP-1R with NNC0640 NAM was obtained from PDB:5VEX [5]. This PDB entry included its own GLP-1R that was positioned very similarly to the one found in PDB:5VAI. Due to this similarity, we chose to use the GLP-1R found in PDB:5VAI in our NAM system and all of the other systems for consistency. To preserve the position of NNC0640, we superimposed the GLP-1R structure in PDB:5VAI onto the one in PDB:5VEX before removing the original GLP-1R chain in PDB:5VEX.

A lipid membrane was needed to create a biologically accurate system and the position of GLP-1R within the membrane was also needed, which was determined using the University of Michigan’s positioning of proteins in membranes (PPM) server [12]. The positioning data was then used to generate a phospholipid bilayer composed of 1-palmitoyl-2-oleoylphosphatidylcholine (POPC) with a 0.15M physiological ion concentration using CHARMM’s membrane builder [13]. This was performed for the apo structure of GLP-1R and for the other systems when GLP-1R was bound to C-1, NNC0460, and GLP-1 with Gs.

Once the lipid membrane was generated for each structure, the files were converted from CHARMM so they would be compatible with the AMBER molecular dynamics package since we used AMBER18 [14]. Further preparation of each system to determine the placement of hydrogens, solvation, charge fitting, periodic boundary conditions, and force field selection for proteins (FF14SB) and lipids (Lipid14) were accomplished using LeAP [15]. The water model used was TIP3P and non-standard molecules, such as our NAM and PAM, were parameterized to generate a coordinate and topology file for MD simulation by using antechamber [16].

**Fig. 2:**
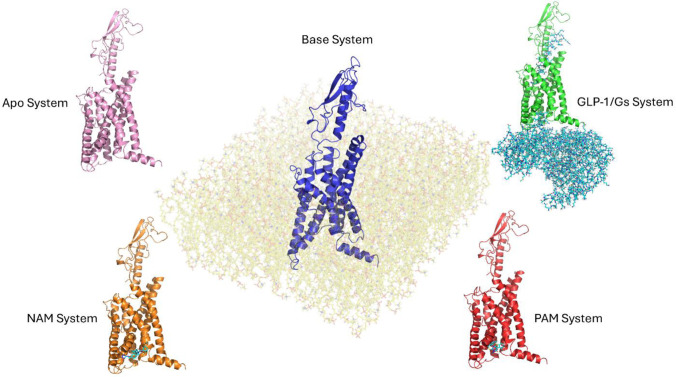
GLP-1R systems with different binding components. The base system of every simulation contains TIP3P waters and K+ and Cl− ions (not pictured for clarity), a POPC lipid bilayer, and a GLP-1R molecule from PDB:5VAI. Additional binding components are GLP-1 with the Gs (green), C-1 in our PAM system (red), and NNC0640 in our NAM system (orange). Our apo system (pink) is identical to the base system (blue) but it undergoes MD simulation while the base system is static and used as a reference

In total, besides a base system that served as a reference starting point, four unique systems were created using the GLP-1R in PDB:5VAI. All systems included GLP-1R, a lipid membrane, waters, and ions as the base. Each system except the apo system differs by what is bound to GLP-1R (Fig. 2).

### MD equilibration and production

Each system, except for the base system, underwent steepest descent minimization under constant volume, heating to 303K under constant pressure, and equilibration until a stable RMSD was obtained before 3 μs of production was performed. The SHAKE algorithm was used to allow a 2 femtosecond timestep and snapshots were taken every nanosecond since that is enough to capture the movement of domains [17]. The shape of each system was cubic and contained approximately 200K to 250K atoms depending on whether the system was just the apo structure within a bilayer with waters and ions or if it contained other additional binding components.

### Analyzing the MD trajectories

The MD trajectories were first analyzed by the root-mean square deviation (RMSD) to quantify the difference that binding components had on GLP-1R during each 3-μs simulation. The GLP-1R in our base system was from PDB:5VAI, which is in an active conformation, and was used as a reference structure for RMSD analysis between the average GLP-1R structure of each system. All translations, rotations, and residues that are not a part of GLP-1R were removed and aligned to reference PDB before generating the average structure with cpptraj [18]. To visualize the 3D structure of the GLP-1Rs and obtain the RMSD values, we utilized PyMOL’s align function with the reference PDB being the target molecule [19]. Backbone atoms (CA, C, N) of GLP-1R were considered during the alignment.

Next, we created protein structure networks (PSNs) for each system by using PSNtools, which uses Wordom as the backend and is a program developed by the same group [20–21]. Before processing the trajectories with PSNtools, we removed all atoms in each system except those that belonged to GLP-1R and aligned those residues to the reference GLP-1R structure in PDB:5VAI using cpptraj.

We utilized most of the default settings used by PSNtools while generating the PSNs for each trajectory, but we read in every frame instead of the default of every 3 frames to capture all changes possible in our simulations, which resulted in 3000 frames being read per system. Two nodes were considered to have a link between them if they were within 4.5 angstroms of each other for a trajectory frame. A highly connected node is considered a hub if it has 4 or more links to it from other nodes or other hubs.

With the PSNs created, we used PSNtools to calculate all of the shortest communication paths between each node within GLP-1R for each system and noted the link and node recurrence of each residue, which is the frequency that links and nodes appear in the calculated pool of shortest paths per frame. We also created an optional label file with a naming scheme that identifies each residue and its location on GLP-1R (ECD, TM1–7, ICL1–3, ECL1–3, or ICD).

The settings used for the calculation of the shortest paths were mostly default also, but we included optional settings, such as including glycines in calculations, a link and hub frequency minimum of 0.5, a meta path recurrence minimum of 0.2, and a correlation value of 0.8. This specified that only links and hubs that are present in 50% of all trajectory frames that have a meta path recurrence rate of 20% and a linear mutual information (LMI) value of 0.8 will be considered in calculating the shortest communication paths.

**Extended Data Figure 1.**
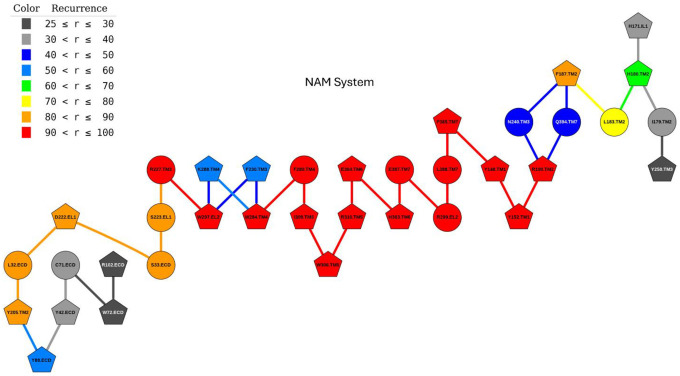
NAM system global meta path

**Extended Data Figure 2.**
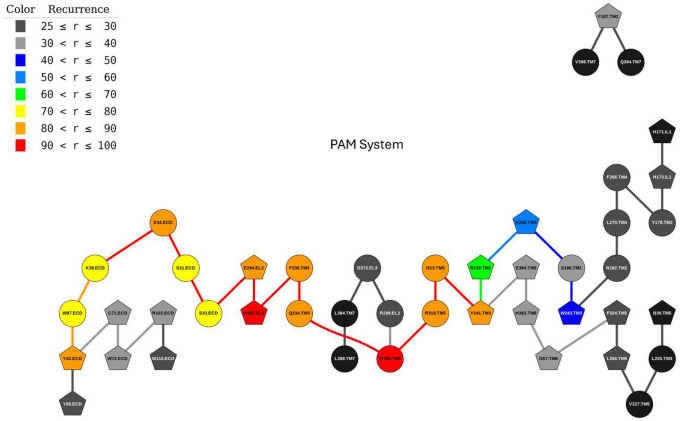
PAM system global meta path

**Extended Data Figure 3.**
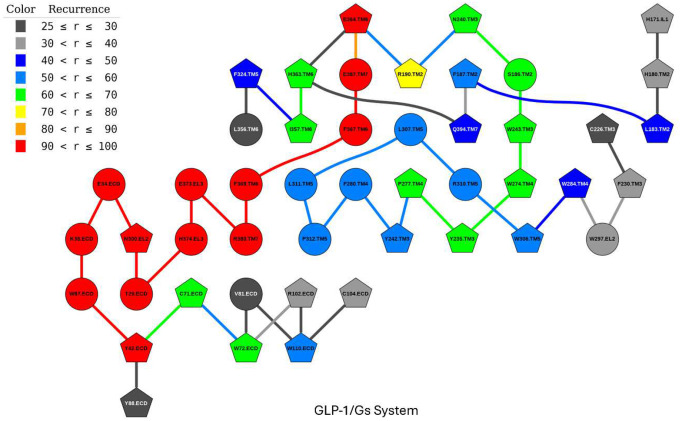
GLP-1/Gs system global meta path

**Extended Data Figure 4.**
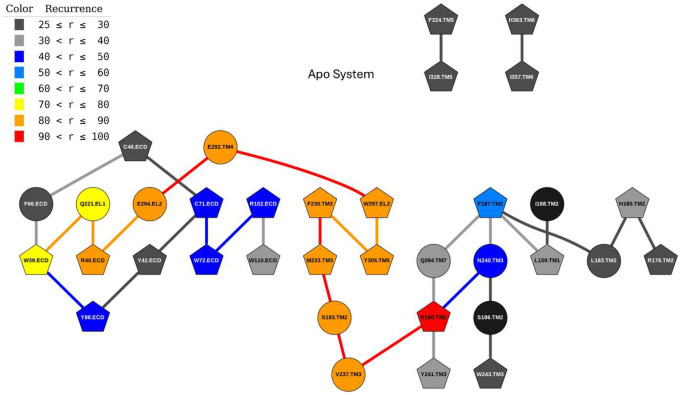
Apo system global meta path

We plotted the global meta path for each system along with a color legend to indicate link and hub recurrence (Extended Data Figures 1–4). The color recurrence legend is the same used in the PSNtools documentation with permission for all four systems.

## Results and Discussion

Employing the first full-length structure of GLP-1R in its active conformation [8], we have created multiple MD systems of GLP-1R under different conditions: the apo structure, the GLP-1/Gs-binding structure, the PAM C-1-binding structure, and the NAM NNC0640-binding structure. Each system was subjected to multi-microsecond molecular dynamics simulations. The simulation trajectory of each system was analyzed and compared using RMSD analysis and PSN theory.

### RMSD comparison between active reference and average structures

**Fig. 3:**
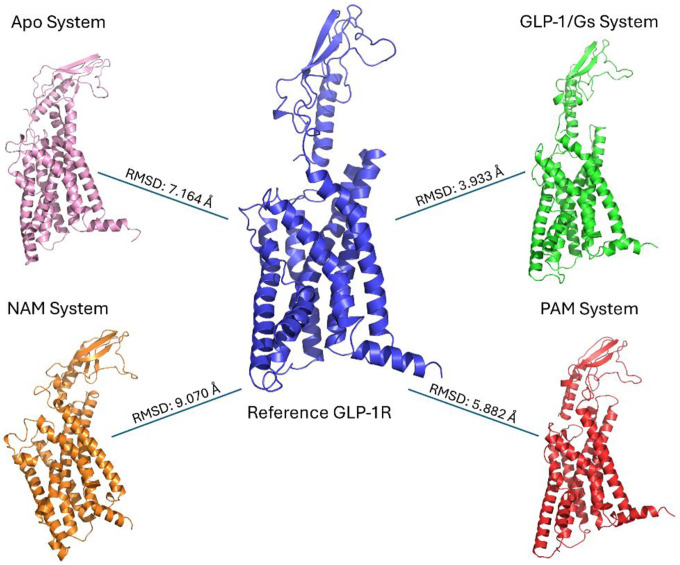
RMSD calculations of average GLP-1R structure compared to reference active state in base system. The reference active state was obtained from GLP-1R in PDB:5VAI since it is classified as being in an active conformation. The average GLP-1R structure within each system was aligned to this reference to quantify changes that occurred due to bound components

RMSD allows us to quantify the structural difference that binding components had on GLP-1R during each 3-μs simulation. The GLP-1R in our base system was from PDB:5VAI, which is in an active conformation, and was used as a reference structure for RMSD analysis between the average GLP-1R structure of each system (Fig. 3). The average receptor from our GLP-1/Gs system has the lowest RMSD value and closest resemblance to the active cryo-EM state. The receptor from our PAM system was the second closest in resemblance, which supports previous findings that C-1 acts as an ago-PAM without the need for GLP-1 to be bound. Our NAM system had the highest receptor RMSD value, which indicates the average receptor structure in this system differs from the activated GLP-1R reference the most. The ECD is also more collapsed in the NAM system when compared to the others and this may reduce accessibility of the orthosteric binding region. The unbound apo system had a receptor RMSD value in between the activated systems and the NAM system, which was expected since it could undergo basal level activation or transition towards the inactive conformation with no binding components.

**Fig. 4:**
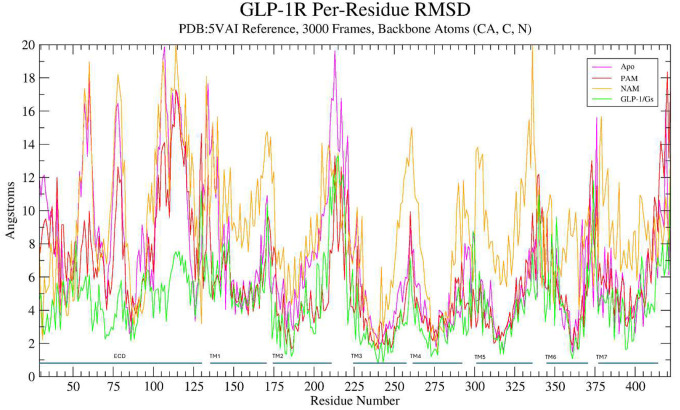
Per-residue RMSD as an effect of binding components. The reference is the active GLP-1R in PDB:5VAI. The residues of the ECD and TM helices are indicated by a blue line.

Further, we also analyzed each system with per-residue RMSD to quantify the changes between the average GLP-1R in each system and the reference GLP-1R (Fig. 4). The per-residue RMSD results also showed that the GLP-1/Gs system had the lowest deviation from the reference structure and the NAM system had the highest. This reflects and reinforces what is seen in the overall RMSD results (Fig. 3) when comparing receptors. Residues of the TM helices are often involved in binding and are reflected as valleys in the plot. The most fluctuations occur in the residues of the ECD and that is expected since these areas are unbound in all systems except GLP-1/Gs.

RMSD analysis demonstrates that different modulators stabilize distinct GLP-1R conformations. Positive effectors, such as GLP-1/Gs and C-1, maintain structures that closely resemble the active cryo-EM state, whereas negative effectors, such as NNC0640 in the NAM system, promote a structurally divergent state that likely shifts towards the inactive state.

### Central polar network analysis

The central polar network is an area that is located above the HETx motif and is important for peptide-mediated activation of GLP-1R [22]. Based on the meta path plot, our NAM system frequently makes links with all of residues in the central polar network.

**Fig. 5:**
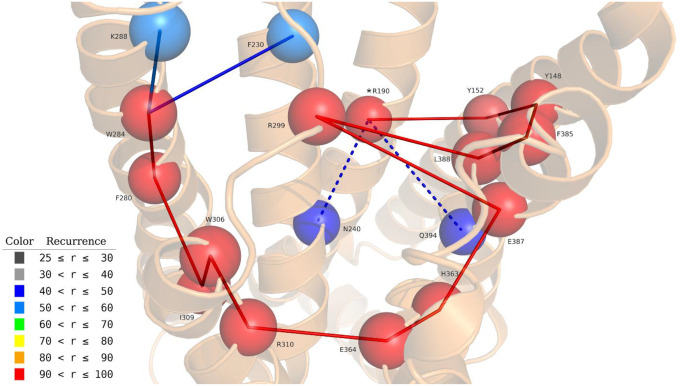
Highly connected central polar network residues in the NAM system. All central polar network residues are highly connected when NNC0640 is bound. Residue R190^2.60b^ on TM2 functions as a starting point as indicated by the asterisk. Solid lines indicate signal propagation in the direction of highest recurrence while dashes indicate signaling in the direction of lower recurrence

When plotting these residues onto the average GLP-1R structure of the NAM system, the residues are concentrated around the GLP-1 binding pocket. This suggests NNC0640 stabilizes a compact arrangement of the central polar network residues that may reduce accessibility of the orthosteric binding region (Fig. 5). Residue R190^2.60b^ is a residue in the central polar network and a hub in all of the global meta paths, which designates it as a highly connected node, so we made it a starting point for the analysis of allosteric signaling. R190^2.60b^ is also connected to N240^3.43b^ with a high recurrence pathway and N240^3.43b^ has been shown to be critical in positive and negative signaling in another study [16].

While only partially depicted, R190^2.60b^ also participates in communication pathways extending towards the intracellular region in the full global meta path (Extended Data Fig. 1). The recurrence levels of residues beyond R190^2.60b^ to N240^3.43b^ or R190^2.60b^ to Q394^7.49b^ are lower than the one depicted for R190^2.60b^ to Y152^1.47b^ signaling towards the ECD so it was omitted for clarity. Signaling towards the ECD appears to be the preferred path for other systems also.

**Fig. 6:**
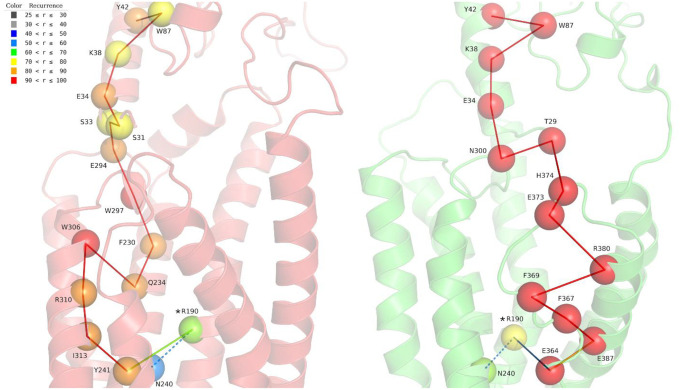
Preferred signaling pathway of the PAM and GLP-1/Gs systems. Allosteric signaling in residues in TM3 and TM5 are preferred when the C-1 PAM is bound (left). Residues in mainly TM6 and TM7 are preferred when GLP-1/Gs are the positive effectors (right). R190^2.60b^ to N240^3.43b^ is the only low recurrence path in both systems. Solid lines indicate direction of highest recurrence and dashes are of the path of lower recurrence

When GLP-1R is bound to positive effectors, such as our C-1 PAM or GLP-1/Gs, there is no continuous link between all residues of the central polar network with high recurrence as when NNC0640 is bound. When compared to the NAM system, there is a different preferred pathway for allosteric signaling (Fig. 6).

Among the highly connected nodes of the PAM system with high recurrence, the highest-recurrence pathway connects R190^2.60b^ to many residues on TM3 and TM5 before recruiting residues in EL2 and the ECD. For the GLP-1/Gs system, highly connected nodes are concentrated more among TM6 and TM7 residues when signaling towards residues in EL3 and the ECD. The residues are not closely clustered as in the NAM system and this can aid in allowing GLP-1 to bind at the orthosteric site above the central polar network.

Both systems also signal downstream from R190^2.60b^ to the intracellular side, but only residues with the highest recurrence towards the ECD are pictured for clarity. Unlike the NAM system, R190^2.60b^ only signals to N240^3.43b^ downstream so it may suggest that signaling is more efficient when positive effectors are bound by narrowing down possible points of divergence from a critical hub like R190^2.60b^.

**Fig. 7:**
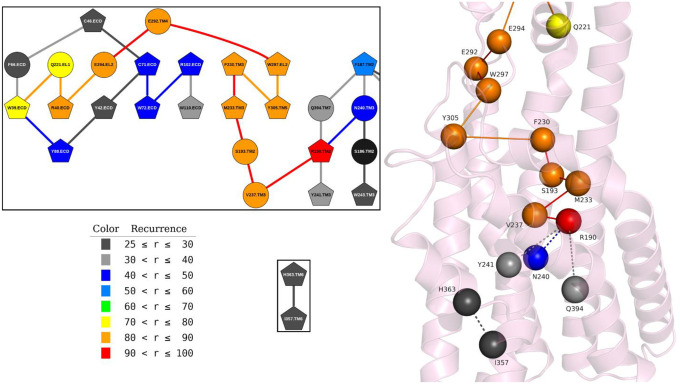
Various meta paths of the apo system. The GLP-1R in the apo system has minimal TM6 nodes that only interact with itself. There are residues on many of the other helices and signaling can occur via many pathways starting from R190^2.60b^. Solid lines indicate direction of highest recurrence and dashes are of the paths of lower recurrence. Nodes are indicated as circles and hubs are indicated as pentagons

When GLP-1R is in its apo state, there are many pathways it can take starting at R190^2.60b^ (Fig. 7) and there is no clustering in the central polar network or strong preference to signal via certain helices like in the other systems. Our apo system only had an unbound GLP-1R and base components that were present in all of our systems, such as waters, ions, and a lipid bilayer present. This served as a baseline meta path and represented how GLP-1R would behave with no bound components in our 3-μs simulation.

The average GLP-1R structure in this system had an RMSD value between those with positive and negative effectors and shared some common residues from both systems when signaling upstream from R190^2.60b^ towards the ECD. When following this path of highest recurrence, it mainly involves residues in TM2, TM3, TM4, and TM5. There is no strong preference towards any TM helices as in the PAM or GLP-1/Gs system.

Residues that belong to TM6 do appear in the meta path of the apo system but there are only two and they do not link to R190^2.60b^ or any other residues except with each other. Interestingly though, the R190^2.60b^ in the apo system can branch out in four directions instead of just two like the other systems. It can signal towards N240^3.43b^, which is a pathway in all systems, towards Q394^7.49b^, like in the NAM system, or towards Y241^3.44b^ or V237^3.40b^, which is uniquely its own. This degree of freedom reflects the flexibility of an unbound receptor and is supported by the meta path analysis.

### HETx motif rearrangement

Certain motifs are conserved and present throughout many GPCRs, such as the HETx motif in GLP-1R. The last residue of the HETx motif may vary depending on the specific GPCR but the motif in our analysis involves H180^2.50b^, E247^3.50b^, T353^6.42b^, and Y402^7.47b^ (HETY). Residue E247^3.50b^ and T353^6.42b^ are located on TM3 and TM6, respectively, and the distance between these two helices can indicate an opening or closing of the G protein site that can enable or prevent activation of GLP-1R [23].

**Fig. 8:**
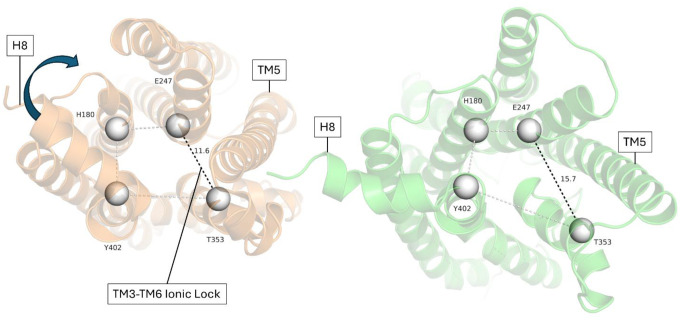
TM3-TM6 distance of HETx motif residues. Residue distances between E247^3.50b^ and T353^6.42b^ are depicted for the average GLP-1R structure in the NAM (left) and GLP-1/Gs system (right). TM5 is pointed towards the transmembrane center in the NAM system and the blue arrow indicates the inward rotation of H8

Instead of choosing which frame in each simulation to take distance measurements with for E247^3.50b^ and T353^6.42b^, we used the average GLP-1R structure created previously for each system since it represents the state the receptor would take on average over each 3-μs simulation. This eliminated any bias in choosing which frames to measure and allowed us to visualize the conformation that GLP-1R would assume as an effect of the different binding components (Fig. 8). The position of other helices, such as TM5 and helix 8, was noted also since they could obstruct molecular binding when compared to an active system.

After aligning each structure to the reference GLP-1R, we found that the TM3-TM6 distance was smallest in our NAM system, which indicates a smaller opening for Gs binding between those helices and possibly an inactive state. The position of TM5 is also pointed more towards the transmembrane center and helix 8 is also rotated inwards when compared to the apo or positive effector systems.

**Extended Data Figure 5.**
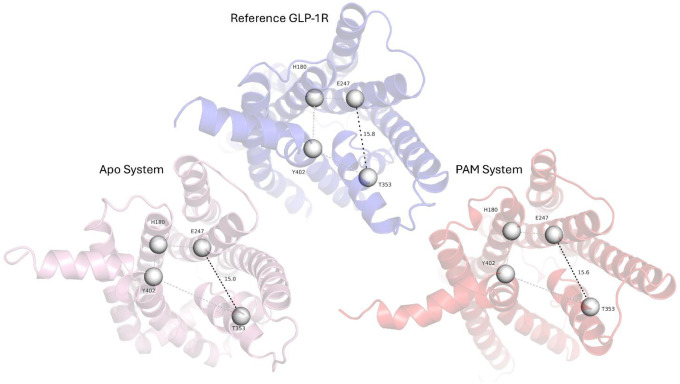
HETx motif TM3-TM6 distance comparisons of remaining systems

This finding supports that NNC0640 acts as a NAM by reducing the distance between E247^3.50b^ and T353^6.42b^ of the HETx motif and by rearranging nearby helices to occlude space and may reduce compatibility with G protein coupling. The reference, apo, and PAM systems that were not depicted had a similar TM3-TM6 distance of 15.8, 15.0, and 15.6 angstroms, respectively, to the 15.7 angstroms observed in the GLP-1/Gs system that is depicted and were omitted for clarity but can be found in Extended Data Figure 5.

When analyzing the full global meta paths, multiple systems contain residues of the HETx motif, specifically H180^2.50b^, but only the NAM system had this residue designated as a hub above the 50% recurrence level with a link to it that is also above 50% (Extended Data Figure 1). This suggests that, while H180^2.50b^ may be a highly connected node in multiple systems, its recurrence in the NAM system is higher and so is its activity level in the HETx motif. Residue E247^3.50b^ was not present in the full meta path, however, and we hypothesize it may be due to being beyond 4.5 angstroms of other short paths or its recurrence was under the threshold specified to be considered.

Nonetheless, the ionic lock distance and meta path hubs suggest that residues of the HETx motif are crucial in maintaining the inactive state of GLP-1R. When positive effectors are bound, signaling through residues of the HETx motif decreases or simply not present at a 20% recurrence level, as with the PAM system (Extended Data Figure 2)

### Analysis of TM2–6-7-H8 polar network

**Fig. 9:**
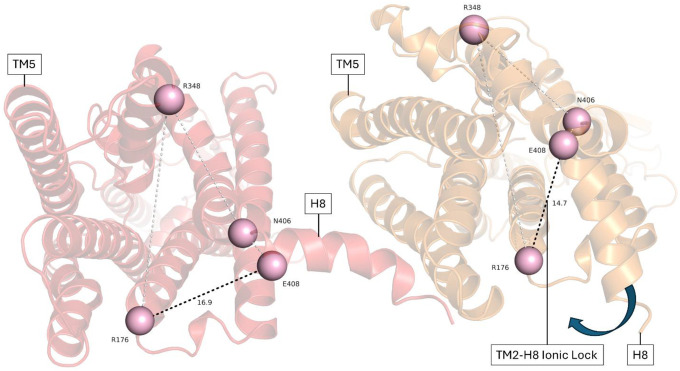
TM2-H8 distance of TM2–6-7-H8 polar network residues. The distance between the TM2-H8 ionic lock of the PAM system (left) is compared to that of the NAM system (right). TM5 and H8 have been labeled to help identify the relative movement towards the center of the transmembrane domain in the NAM system when compared to the PAM system. This inward movement is unique to the NAM system and the other systems were not depicted for clarity.

The TM2–6-7-H8 polar network is similar to the HETx motif in that it also has an ionic lock that can inhibit GLP-1R activation. This lock is between TM2 and H8, however, and is located further down on the cytoplasmic side of GLP-1R. As in the HETx motif rearrangement, we found that TM5 also points more toward the center of the transmembrane domain and H8 rotates inwardly more in the NAM system than in the other systems when analyzing this network of residues (Fig. 9)

**Extended Data Figure 6.**
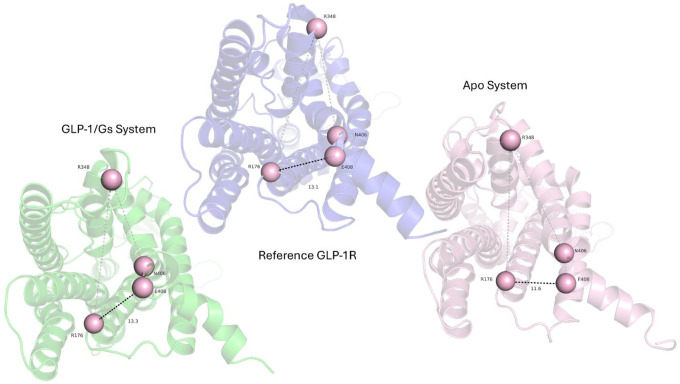
TM2–6-7-H8 network distance comparisons of remaining systems

The distance measurement of the TM2-H8 ionic lock in the NAM system is smaller than that of the PAM system. However, it was not the smallest overall like in the TM3-TM6 ionic lock of the HETx motif and the smallest TM2-H8 ionic distance belongs to the apo system (Extended Data Figure 6). This suggests that other factors like rotation of helices towards the transmembrane, which is only noticeably present in the NAM system, may play a larger role in occluding space from that region.

The binding of molecules at the cytoplasmic end also requires space, whether it be a NAM, PAM, or the Gs G protein, and the apo system does not have any bound effectors. Since nothing is bound, this could also explain why the apo system is able to have the smallest TM2-H8 ionic lock distance and this lock may be more important in inhibiting GLP-1R activation in the apo system than in the NAM system.

When analyzing the meta paths for all of the systems, the apo system was the only system that included residues of the TM2–6-7-H8 network, specifically R176. Even though the recurrence level is low, R176 is still a hub so it is a highly connected node and its contribution to the meta path supports its ability in maintaining the ionic lock distance when compared to the other systems.

### Implications and Future Perspectives

Molecular dynamics and modeling software allowed us to take a static structure and build a solvated bilayer system upon it to evolve through time and space. With the data obtained through these simulations, network theory allowed us to visualize the residues involved in allosteric signaling pathways.

From computing the average GLP-1R structures in our NAM system, we were able to ascertain that the TM3-TM6 distance is smallest when NNC0640 is bound, which can inhibit the activation of GLP-1R. The rearrangement of TM5 and H8 in the HETx motif and the TM2–6-7-H8 polar network of this system also showed movement towards the transmembrane center to occlude space. Mapping the nodes and hubs obtained through network theory to the average GLP-1R structure in the NAM system showed us many residues of the central polar network are involved in the signaling pathway and clustered close together when bound to a negative effector and may reduce accessibility of the orthosteric binding region.

Comparison of the average GLP-1R structures with the active reference PDB showed us that the NAM system had the greatest RMSD variation when compared to apo and positive effector systems. Mapping the nodes and hubs from to the average GLP-1R in the PAM and GLP-1/Gs system showed us that there is strong preference to signal towards the ECD in both cases and the PAM system preferred to recruit residues on TM3 and TM5 and the GLP-1/Gs system preferred residues in TM6 and TM7. The apo structure involved residues in TM2, TM3, TM4, and TM5 when taking the path with highest recurrence. TM6 residues in the meta path of the apo system are minimal and may reflect occasional basal level activation.

Taken together, these results suggest that activation and inhibition of GLP-1R arise from distinct allosteric communication networks rather than solely distinct conformational states. Positive and negative modulators selectively rewire residue-level communication pathways, with a conserved R190^2.60b^-N240^3.43b^ communication axis present across all systems. A summary of key observations is included below (Table 1).

In the future, we may plan to simulate other GPCRs with bound components to elucidate pathways that are currently unknown. The meta paths used in this study had no biasing and we were able to detect residues commonly found in experimental studies with molecular dynamic trajectories and network theory. We are confident that this can be done with other GPCRs and can help validate experimental data and uncover new pathways.

## Extended Data

## Figures and Tables

**Table 1.** Summary of key observations and measurements with average GLP-1R structure in each system

System	Average GLP-1R RMSD	TM3-TM6 Distance in HETx Motif	TM2-H8 Distance in TM2–6-7-H8	Avg. GLP-1R TM5 and H8 Positions	High Recurrence Pathway	Observed Structural State
NAM	9.07 Å	11.6 Å	14.7 Å	Towards TM center	Central Polar Network	Inactive-like
Apo	7.16 Å	15.0 Å	11.6 Å	Away from TM center	Mixed	Intermediate-like
PAM	5.88 Å	15.6 Å	16.9 Å	Away from TM center	TM3/TM5	Active-like
GLP-1/Gs	3.93 A	15.7 Å	13.3 Å	Away from TM center	TM6/TM7	Active-like
